# Extracellular metabolite production by *Bacteroides* strains varies depending on carbohydrates used as carbon sources

**DOI:** 10.1128/aem.00542-26

**Published:** 2026-07-21

**Authors:** Yi-Hsuan Lee, Verena Siewers, Johan Larsbrink

**Affiliations:** 1 Division of Industrial Biotechnology, Department of Life Sciences, Chalmers University of Technology 684649, Gothenburg, Sweden; 2 Division of Systems and Synthetic Biology, Department of Life Sciences, Chalmers University of Technology 684649, Gothenburg, Sweden; University of Nebraska-Lincoln, Lincoln, Nebraska, USA

**Keywords:** gut microbiota, carbohydrate utilization, SCFA, short-chain fatty acid, *Bacteroides thetaiotaomicron*, *Bacteroides*

## Abstract

The microorganisms comprising the gut microbiota perform various important functions, such as producing short-chain fatty acids (SCFAs) and other metabolites that can be taken up by the host and used as energy sources or as signaling molecules. A major nutrient for the microbiota is dietary fiber, mainly composed of complex polysaccharides that cannot be degraded by host enzymes. Species from the Bacteroidota phylum are regarded as proficient carbohydrate degraders, partly thanks to their multi-gene systems, known as polysaccharide utilization loci (PULs), encoding the necessary proteins for carbohydrate capture and cleavage. Linking which species can grow on which polysaccharides, depending on the presence of corresponding PULs encoded in their genomes, has been studied intensely in the last decades. However, little is known about which metabolites they produce from carbohydrate consumption, as previous investigations have mainly used glucose as the sole carbon source. In this study, we grew several Bacteroidota species on different carbohydrates, from monosaccharides to polysaccharides, and quantified the resulting extracellular metabolites produced. The results reveal different extracellular metabolite profiles, both intraspecies and interspecies, depending on the carbon sources. Surprisingly, in some cases, the production of extracellular metabolites differed between growth on a homopolysaccharide and its constituent monosaccharide. The results indicate that the metabolite output of gut species is not static but varies with the nutrients present, adding to the complexity of the interplay among dietary fiber, microbial ecology, and gut health.

**IMPORTANCE:**

The human gut microbiota is closely linked to health, and metabolism of different types of dietary fibers is a core activity within this community. Many studies have investigated the composition of the microbiota and sought to relate the presence and abundance of species to health or disease, often attributing a genus or species to consumption or production of a certain metabolite to explain an overall phenotype. It is known that bacteria have different carbohydrate-degrading abilities, but they may not produce a static output of metabolites from the consumption of different carbohydrates, from monosaccharides to complex polysaccharides. This study demonstrates that common bacteria in the *Bacteroides* genus may drastically alter their production of extracellular metabolites. The findings add another layer of complexity to the dynamics of the gut environment and are important for better understanding the interplay among diet, the microbiota, and the host.

## INTRODUCTION

The gastrointestinal tract represents a rich community of diverse microorganisms that predominantly rely on the host’s diet for nutrients. In mammalian gut microbiomes, dietary fiber represents an important nutrient source that is converted by microbial metabolism into products such as organic acids, which can, in turn, be taken up by host cells and provide multiple health benefits (1). The phylum Bacteroidota is often a dominant part of the human gut microbiome, and its species are regarded as efficient consumers of complex carbohydrates, such as plant- or host-derived polysaccharides, and the proportions of species correlate with the host’s particular diet (2–4). Several studies have focused on the carbohydrate-utilization abilities of Bacteroidota species, which are often linked to the so-called polysaccharide utilization loci (PULs) in their genomes—multi-gene loci encoding all necessary proteins for carbohydrate capture, transport, and enzymatic cleavage (2, 5–8). Especially species from the *Bacteroides* genus have received much attention, and detailed studies combining biochemistry, biophysics, and genetics have shed light on how PULs can enable deconstruction of glycans such as plant hemicelluloses (3, 5, 8), pectin (9), microbial glycans (7), and even host mucin (10, 11). Each PUL targets a specific glycan type, and the repertoire of PULs encoded by a species thus affects its nutritional niche as well as “preferences” for, or prioritization of, different polysaccharides encountered in the competitive gut environment (2, 12). This, in turn, leads to different abundances of species based on the host’s nutrient intake (13–15).

After cleavage of complex oligo- and polysaccharides into their respective monosaccharide building blocks, these are further metabolized through different pathways to support growth, which, apart from the generation of energy and biomass, also results in the production of different secreted metabolites (16–19). Short-chain fatty acids (SCFAs) or other organic acids (OAs) present in the human gut have been widely reported and studied for their correlation with various diseases and overall health of the gut epithelial cells (20, 21). *Bacteroides* and related species are typically reported to produce a range of organic acids, with acetate, propionate, and succinate as major end products (22), but lactate, formate, and malate may also be produced in non-negligible amounts (17, 23, 24). While it is known that many *Bacteroides* species can grow on multiple monosaccharides (3), most *in vitro* studies that have investigated metabolite profiles have utilized glucose as the carbon source (17, 25). Monosaccharide fermentation is expected to lead to the same downstream precursor molecule, phosphoenolpyruvate (PEP), which is then converted to SCFAs or other metabolites (26). In a recent study by Keitel et al., *Phocaeicola vulgatus* (formerly *Bacteroides vulgatus*) was grown in different conditions, including with a range of mono-, oligo-, and polysaccharides as carbon sources (27). The different carbohydrates resulted in different growth behaviors, such as differences in lag phase length or final culture density (OD_600_), but no major differences were seen in the extracellular metabolite output profiles when growing the bacterium on individual mono-, oligo-, or polysaccharides, and the metabolites consisted mainly of acetate, succinate, and lactate.

Gut Bacteroidota species are expected to encounter and metabolize a wide range of carbohydrates, but there is still limited information on whether this leads to static or varied outputs of extracellular metabolites. Here, our aim was to broadly determine whether the extracellular metabolite output from select Bacteroidota species would be consistent or variable depending on the carbohydrate carbon source. Using a combination of growth studies and metabolite and carbohydrate analyses, we demonstrate that the production of organic acids can differ greatly from that observed from glucose-grown cultures. This information adds to the complexity of nutrient metabolism and potential cross-feeding in the gut environment, and consequently to the understanding of how diet affects overall gut health.

## MATERIALS AND METHODS

### Strains, media, and growth conditions

The strains used in this study, *Bacteroides thetaiotaomicron* VPI-5482, *Bacteroides ovatus* ATCC 8483, *Bacteroides cellulosilyticus* WH2, *Bacteroides eggerthii* ATCC 27,754, *Phocaeicola vulgatus* ATCC 8482, and *Dysgonomonas mossii* CCUG 43457T, were routinely grown in tryptone-yeast-glucose (TYG) medium (10 g/L tryptone, 5 g/L yeast extract, 4 g/L glucose, 100 mM potassium phosphate [pH 7.2], 15 mM NaCl, 8.5 mM [NH_4_]_2_SO_4_, 4 mM l-cysteine, 200 mM l-histidine, 100 mM MgCl_2_, 72.1 mM CaCl_2_, 1.9 mM hematin, 1.4 mM FeSO_4_∙7H_2_O, 1 mg/mL vitamin K_3_, and 5 ng/mL vitamin B_12_). For cultivation on different carbon sources, a minimal medium (MM; 100 mM KPO_4_ [pH 7.2], 15 mM NaCl, 8.5 mM [NH_4_]_2_SO_4_, 4 mM l-cysteine, 1.9 µM hematin, 200 µM l-histidine, 100 µM MgCl_2_, 1.4 µM FeSO_4_∙7H_2_O, 50 µM CaCl_2_, 1 mg/L vitamin K_3_, and 5 µg/L vitamin B_12_) was used with 5 g/L carbon source. The strains were grown anaerobically under static conditions at 37 °C in an anaerobic chamber (Coy Laboratory Products) using a gas mix of 10% H_2_, 10% CO_2_, and 80% N_2_. For small-scale cultivations, cells were grown in 96-well plates in a Sunrise microplate reader (Tecan), internal temperature at 36 °C, with the optical density at 600 nm (OD_600_) measured every 10 min up to 6 days. Overnight cultures were washed twice in 2× MM without a carbon source before being resuspended 1:50 in the same medium, and 100 µL of the resuspended cells was added to 100 µL of 10 g/L carbon source in a 96-well plate, in at least triplicate. The carbon sources used were ᴅ-glucose (Merck), ᴅ-galactose (Sigma), ᴅ-maltose (Merck), ᴅ-mannose (Thermo Scientific Inc.), ᴅ-fructose (Thermo Scientific Inc.), ᴅ-arabinose (Sigma), ᴅ-xylose (Sigma), l-fucose (Biosynth), l-rhamnose (Sigma), *N*-acetylglucosamine (Sigma), ᴅ-glucuronic acid (Merck), ᴅ-galacturonic acid (Merck), ᴅ-lactose (Sigma), sucrose (Merck), ᴅ-raffinose (Sigma), maize amylopectin (Sigma), sugar beet arabinan (Megazyme), larch arabinogalactan (Biosynth), chondroitin sulfate (sodium salt, Sigma), carob galactomannan (Megazyme), konjac glucomannan (Megazyme), levan (Megazyme), citrus pectin (Sigma), polygalacturonic acid (Megazyme), rhamnogalacturonan I (Megazyme), potato starch (Sigma), and tamarind xyloglucan (Megazyme). The carbohydrates were sterilized either by autoclaving or filter sterilization.

### Sampling of extracellular metabolites

For collection of extracellular metabolites, cultures were scaled up to 2 mL in loosely capped glass culture tubes in the same anaerobic conditions as described earlier. Cultures were inoculated to a starting OD_600_ between 0.05 and 0.1 (Biochrom Colourwave CO7000 Medical Colorimeter; VWR International), and growth was manually monitored to determine appropriate sampling time points. The average culture time was 2–3 days, and samples were collected at three time points: the end of the exponential phase just before reaching the maximum OD_600_, at the maximum OD_600_, and 2–3 h after the growth maximum. In total, 500 µL samples were centrifuged for 3 min at 20,000 × *g*, after which the supernatant was collected and stored at −20 °C prior to analysis.

### Metabolite and carbohydrate analyses

The collected metabolites were analyzed by high-performance liquid chromatography (HPLC), using an LC-4000 instrument (JASCO). Chemicals used for preparation of standards included: α-ketoglutarate (Sigma), acetate (Acros Organics), butyrate (Larodan AB), ethanol (Fisher Scientific), formate (Acros Organics), lactate (Sigma), malate (Merck), propionate (Acros Organics), pyruvate (Gibco), and succinate (Merck). The samples were filtered (0.22 µm, nylon; GVS Filter Technology) into HPLC vials prior to analysis. The samples were separated using a 300 × 7.8 mm Rezex ROA-Organic Acid H^+^ (8%) column (Phenomenex), held at 80 °C under isocratic conditions with 5 mM H_2_SO_4_ as the eluent. Metabolites were detected using UV at 210 nm or refractive index (RI) and quantified against standard curves using reference compounds (Fig. S1), as described previously (17).

Remaining carbohydrates in the samples were analyzed using high-performance anion-exchange chromatography with pulsed amperometric detection (HPAEC-PAD) using a Dionex ICS-5000 system (Thermo Fisher Scientific Inc.) operated by Chromeleon v.7.2.10 (Thermo Fisher Scientific Inc.). As for HPLC, the samples were filtered prior to peak analysis. Carbohydrates were separated using either a 2 × 250  mm Dionex Carbopac PA1 column for monosaccharides, or a PA200 column for di- and oligosaccharides (Thermo Fisher Scientific Inc.) with a 2 × 50  mm guard column, respectively. The column temperature was 25 °C for PA1 and 30 °C for PA200. The eluents were A—water, B—300 mM NaOH, and C—100 mM NaOH and 85 mM sodium acetate for the PA1 column; 1 M NaOH and 100 mM sodium acetate for the PA200 column. The samples were eluted using eluent A and detected after post-column addition of eluent B. Thereafter, the column was washed with eluent C and equilibrated with eluent A. Analytes were identified and quantified against pure standards. For statistical analyses, Student’s T-tests with two-tailed distribution and two-sample unequal variance (heteroscedastic) were employed.

## RESULTS AND DISCUSSION

### Selection and cultivation of Bacteroidota species for sampling extracellular metabolites

Six different Bacteroidota species were selected based either on their carbohydrate utilization having been well-studied—*Bacteroides thetaiotaomicron* (*B. theta*) (3), *Bacteroides ovatus* (3), *Bacteroides cellulosilyticus* (28), *Phocaeicola vulgatus*—or less studied but with potential capability for growth on a range of carbohydrates*—Bacteroides eggerthii* (29, 30) and *Dysgonomonas mossii* (31, 32). Other species regarded as biosafety level 2, such as *Bacteroides fragilis*, were furthermore excluded. The growth of the selected species was first assessed in 96-well plates on various carbohydrates as the sole carbon source, from mono- to oligo- and polysaccharides. As expected, *B. theta*, *B. ovatus*, and *B. cellulosilyticus* were able to grow well on a wide range of these, reaching OD_600_ values above 1 in <48 h, while the carbohydrate utilization of the other three species was limited, especially on more complex substrates, and they were therefore excluded from further experiments (Fig. S2). Potentially, optimization of growth media and conditions could facilitate future investigation of these species as well, to provide broader information on the phylum outside the genus *Bacteroides*.

**Fig 1:**
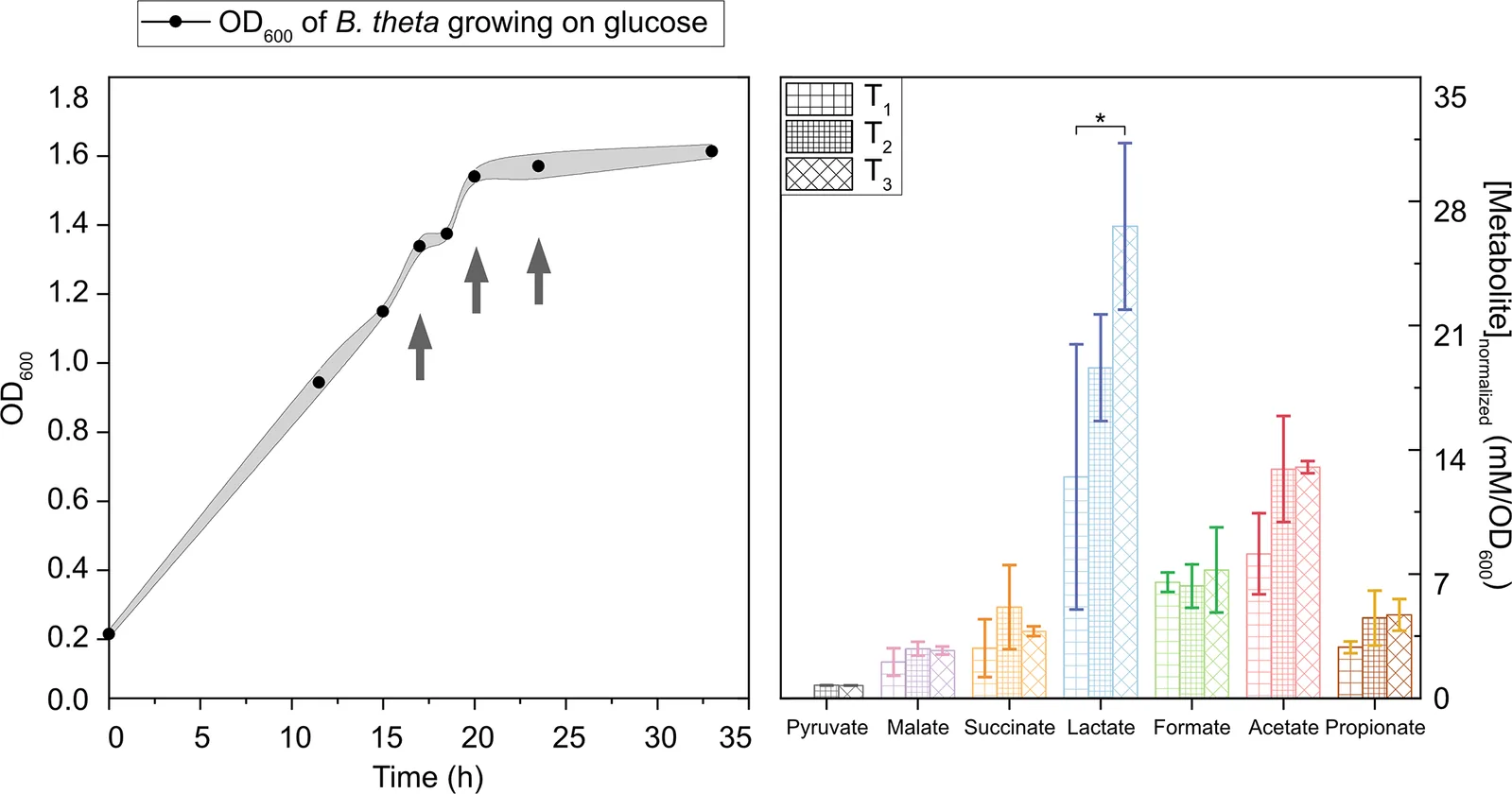
The growth curve of *Bacteroides thetaiotaomicron* grown on glucose (left) and the corresponding metabolite output (right). T_1_, T_2_, and T_3_ indicate the timepoints for sampling and are shown as arrows on the growth curve, representing the stages of pre-maximum, maximum OD_600_, and post-maximum in cell growth. Data were taken from at least triplicate cultures in MM with an initial glucose concentration of 5 g/L, and metabolite concentrations are normalized to the measured OD_600_. Shading represents standard deviation. A Student’s T-test was conducted for the lactate production among the three sampling points, showing a significant difference between T_1_ and T_3_ (*, *P*-value ≤ 0.01), while no significant difference was seen between T_2_ and T_3_.

The growth of *B. theta*, *B. ovatus*, and *B. cellulosilyticus* was then assessed in scaled-up 2-mL vial cultures to facilitate subsequent sampling and metabolite analysis. *Bacteroides* growth curves typically consist of a lag phase, exponential growth to an OD_600_ peak, sometimes followed by a slight drop, and then a stable post-exponential phase before cell death (33, 34). To determine whether the metabolite production was affected by the growth phase, samples were taken from cultures with glucose (Glc) as carbon source from the late exponential phase, at the maximum OD_600_, and 2–3 h after this maximum (Fig. 1). In these larger cultures, a distinct sharp OD_600_ peak was not observed, but rather a stable maximum after the onset of the stationary phase. No significant changes in metabolite proportions were seen between the later sampling timepoints, similar to previous studies of *P. vulgatus* (27). The timepoint at which the OD_600_ maximum was reached was chosen as the main sampling point for metabolite analysis to achieve higher concentrations than in the early exponential phase and to limit the risk of lysis products in the stationary phase.

### Extracellular metabolites produced by *Bacteroides thetaiotaomicron* varied when grown on different monosaccharides

**Fig 2:**
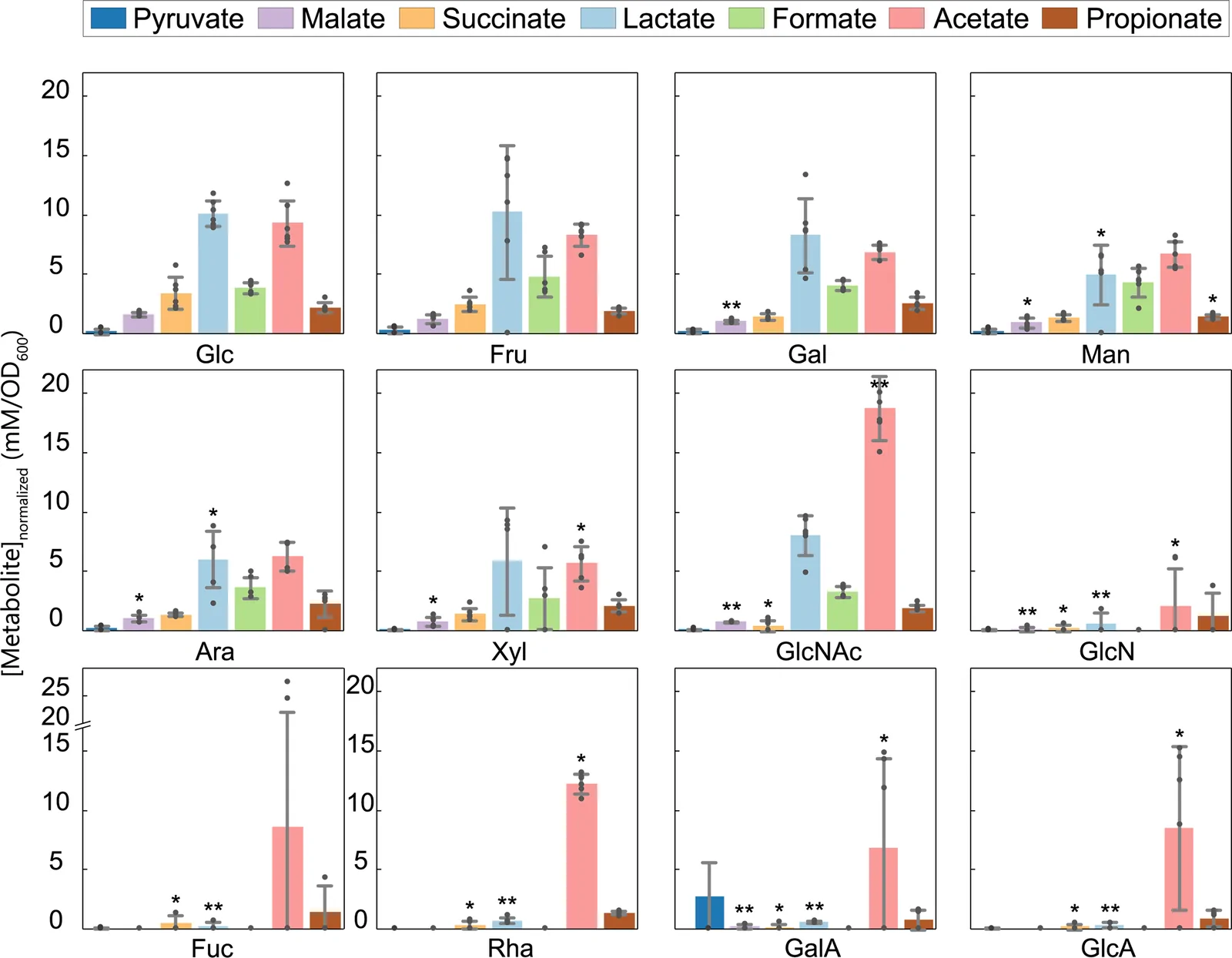
Extracellular metabolites detected from cultures of *Bacteroides thetaiotaomicron* using various monosaccharides as carbon sources. All carbohydrates had an initial concentration of 5 g/L. The concentration of extracellular metabolites was quantified using HPLC, from sampling at the T_2_ timepoint, and normalized to the OD_600_ values, which were in a similar range (>1; Data S1). Individual data points are shown; the bars show the mean value, and error bars represent standard deviation from at least triplicate experiments. Asterisks indicate *P*-values from Student’s T-tests comparing the amount of each metabolite to what was produced from Glc, with * for *P* ≤ 0.01 and ** for *P* ≤ 0.001.

When *B. theta* was grown on Glc, it produced the expected set of metabolites: mainly acetate and lactate (17), a lower amount of formate, and small amounts of malate, propionate, and succinate (Fig. 2). The proportions differed somewhat from previous studies (17), possibly due to the larger cultivation volumes and longer duration of growth. Next, *B. theta* was grown on 11 other monosaccharides, including neutral hexoses: galactose (Gal), mannose (Man), and fructose (Fru); pentoses: arabinose (Ara) and xylose (Xyl); aminosugars: *N*-acetyl glucosamine (GlcNAc) and glucosamine (GlcN); uronic acids: glucuronic acid (GlcA) and galacturonic acid (GalA); and deoxysugars: rhamnose (Rha) and fucose (Fuc). In all cultures, the final OD_600_ reached above 1, and there were no significant remaining carbohydrates left in the culture media (Tables S1 and S2). On the neutral hexoses and pentoses, the proportions and amounts of extracellular metabolites were rather similar to Glc, with mainly formate, acetate, and lactate detected, though the profiles were not identical (Fig. 2; Data S1). Significant differences (*P*-values < 0.01) compared to Glc were observed most commonly for malate production, i.e., on Gal, Ara, and Xyl, and for Man, both lower lactate and malate production were observed. The results overall suggest that the metabolism of these neutral sugars, both hexoses and pentoses, that go through typical import, isomerization, phosphorylation, and epimerization pathways (26, 35, 36), has minor effects on metabolic output. Among the aminosugars, the profiles differed more clearly from Glc, and especially for GlcNAc, a high concentration of acetate was observed, possibly stemming from the acetyl groups on the monosaccharide being cleaved off and exported rather than being further metabolized. Increased acetate concentrations have been shown to inhibit *B. theta* metabolism (37), with slowed growth resulting from externally added acetate, whereas growth on GlcNAc was rapid in our setup (Table S1). The reduced production of succinate may be a result of increased production of CO_2_ (37), though analyses to confirm this hypothesis were not performed. A similar increase in produced acetate was not observed for GlcN, which lacks this acetyl group, and curiously, with this sugar as a carbon source, very low metabolite formation was seen, and no formate was detected. In continuous cultures, similar low metabolite production from GlcN has been shown (38). Potentially, GlcN alone does not induce high enough upregulation of metabolizing genes, such as *nagB* (encoding glucosamine-6-phosphate deaminase) (39), which might induce a general stress response. With the two deoxysugars (Fuc and Rha) and the uronic acids (GlcA and GalA), acetate was the clear main product, though with considerable variation among the biological replicates, and in these cultures only small amounts of other metabolites were detected. Uronic acids are metabolized via the isomerase (Ashwell) pathway (40), which may explain the altered metabolic output. Fuc, on the other hand, may be linked to nutrient-limiting conditions for *B. theta* (41); possibly, both deoxysugars lead to reduced overflow metabolism and potential nutrient sharing/cross-feeding (41, 42).

### Extracellular metabolites produced by *Bacteroides thetaiotaomicron* during growth on oligo- and polysaccharides

**Fig 3:**
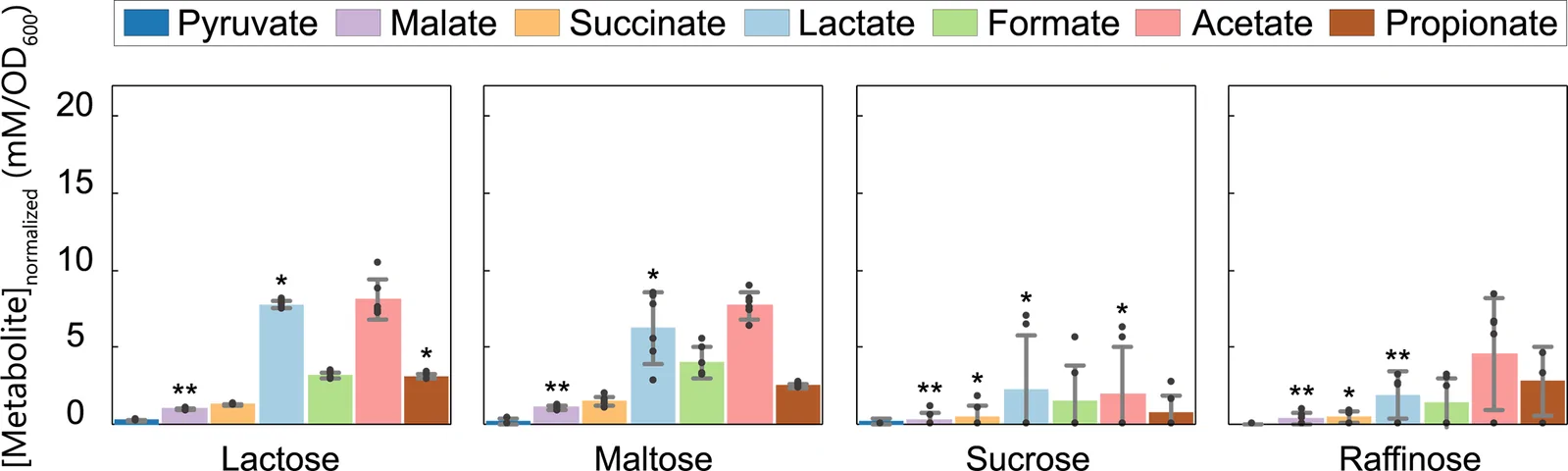
Extracellular metabolites detected from cultures of *Bacteroides thetaiotaomicron* using oligosaccharides as carbon sources. The sampling was done as for the monosaccharides; cultures were grown with 5 g/L carbohydrates as starting carbon source and samples collected at the T_2_ timepoint, and metabolites monitored using HPLC. The concentrations are normalized to the OD_600_ values, which were all in a similar range (around 1.5; Data S1). Individual data points are shown; the bars show the mean value, and error bars represent standard deviation from at least triplicate experiments. Asterisks indicate *P*-values from Student’s T-tests comparing the amount of each metabolite to what was produced from Glc, with * for *P* ≤ 0.01 and ** for *P* ≤ 0.001.

Next, the metabolite production profiles during growth on oligo- and polysaccharides were monitored in the same manner as for the monosaccharides. On the oligosaccharides, maltose (Mal; Glc-α[1→4]-Glc), lactose (Lac; Gal-β[1→4]-Glc), sucrose (Suc; Glc-α[1↔2]-β-Fru), and raffinose (Raf; Gal-α[1→6]-Glc-α[1↔2]-β-Fru; galactosylated sucrose), the growth rate was slower than on Glc, except for Mal, but the final OD_600_ reached similar values(~1.5) in all cultures. To verify that the carbohydrates had been consumed, HPAEC-PAD was used to analyze both the oligosaccharide- and monosaccharide building block concentrations at the end of growth, which showed that the carbon sources had been fully consumed except in the lactose and raffinose cultures, where trace amounts still existed at the time of sampling (<4% of starting amount; Tables S1 and S2). To better understand the metabolite profile/production per cell, the measured metabolite concentrations were all normalized to the maximum OD_600_ measured at the sampling timepoint (T_2_; Fig. 1). The OA production profiles showed a similar pattern to that of Glc (Fig. 3), as could be expected from their respective hexose building blocks, but with differences in concentrations, such as slightly lower concentrations of lactate and malate overall and interestingly a noticeable lower concentration of produced metabolites from sucrose and raffinose. The generally lower metabolite concentrations could be attributed to the energy expenditure for production of glycoside hydrolases to cleave the oligosaccharides to monosaccharides (Fig. 2). It should be noted that the metabolite production from oligo- and polysaccharides was for some cultures quite variable, where certain metabolites were produced in significant amounts in some samples and not detected in others.

**Fig 4:**
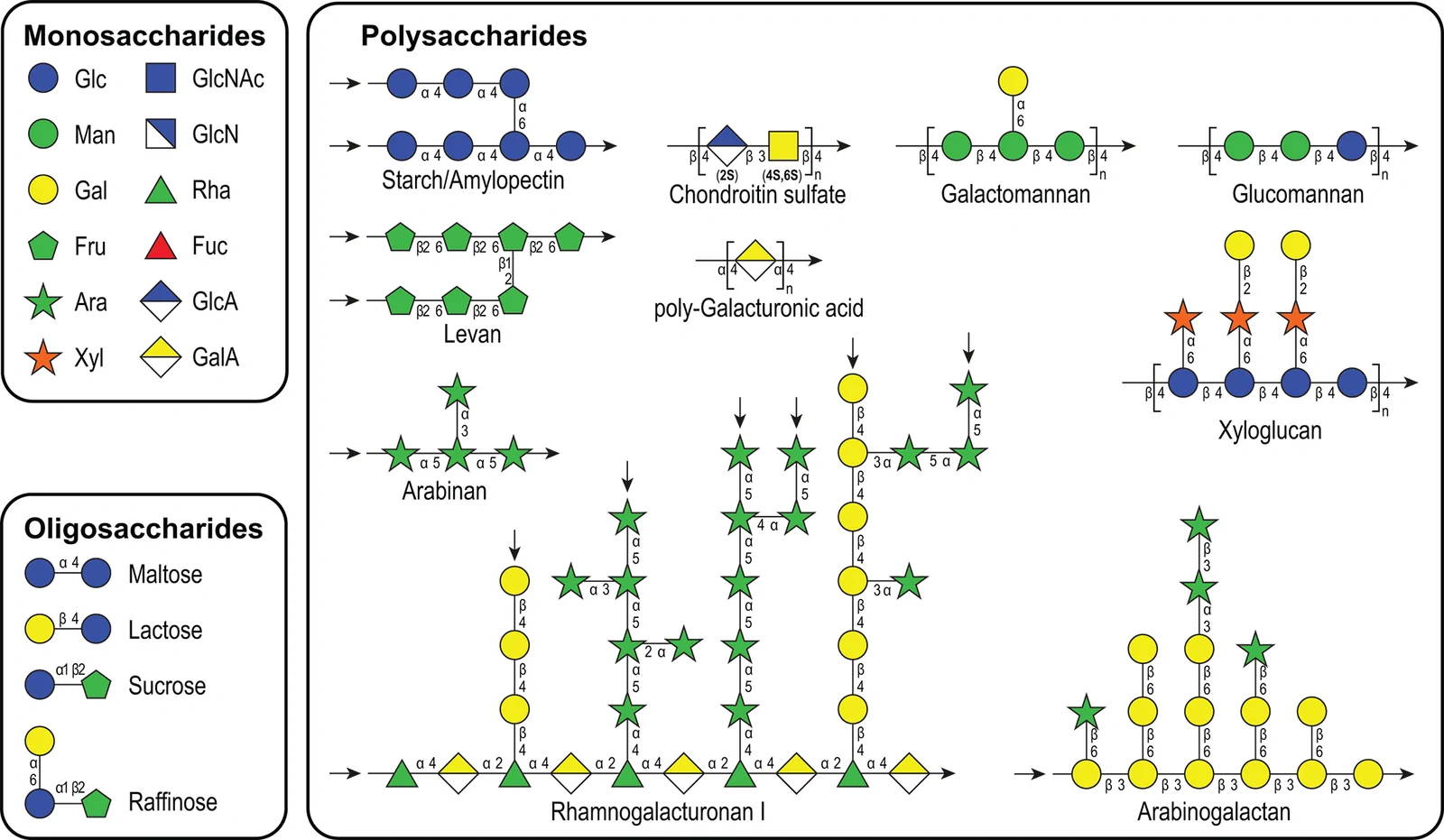
Oligo- and polysaccharide structures. Structures of the oligosaccharides and representative structures of the polysaccharides used in this study, marking the different linkages and monosaccharide building blocks.

When polysaccharides were used as carbon source, unsurprisingly, *B. theta* did not grow on the hemicelluloses galactomannan (GM), (konjac) glucomannan (KGM), or xyloglucan (XG) (3), but clear growth was observed on the glycosaminoglycan chondroitin sulfate (CS), fructose-based levan, the pectic polysaccharides arabinogalactan (AG), arabinan, rhamnogalacturonan I (RGI), polygalacturonic acid (pGalA), and citrus pectin, as well as starch and amylopectin, with final OD_600_ values in similar ranges, above 1 (for monosaccharide building blocks and linkages of oligo- and polysaccharides, see Fig. 4). For certain polysaccharides, the time to reach the growth maximum was rapid, such as arabinan and pGalA, indicating efficient depolymerization and metabolism of these more complex glycans, while the growth was slower on others such as citrus pectin, amylopectin, and starch where the cultures took >40 h to reach the OD_600_ maximum (Table S1). As for the oligosaccharides above, metabolite concentrations were normalized to the OD_600_ values at the sampling point to analyze production relative to cell number.

**Fig 5:**
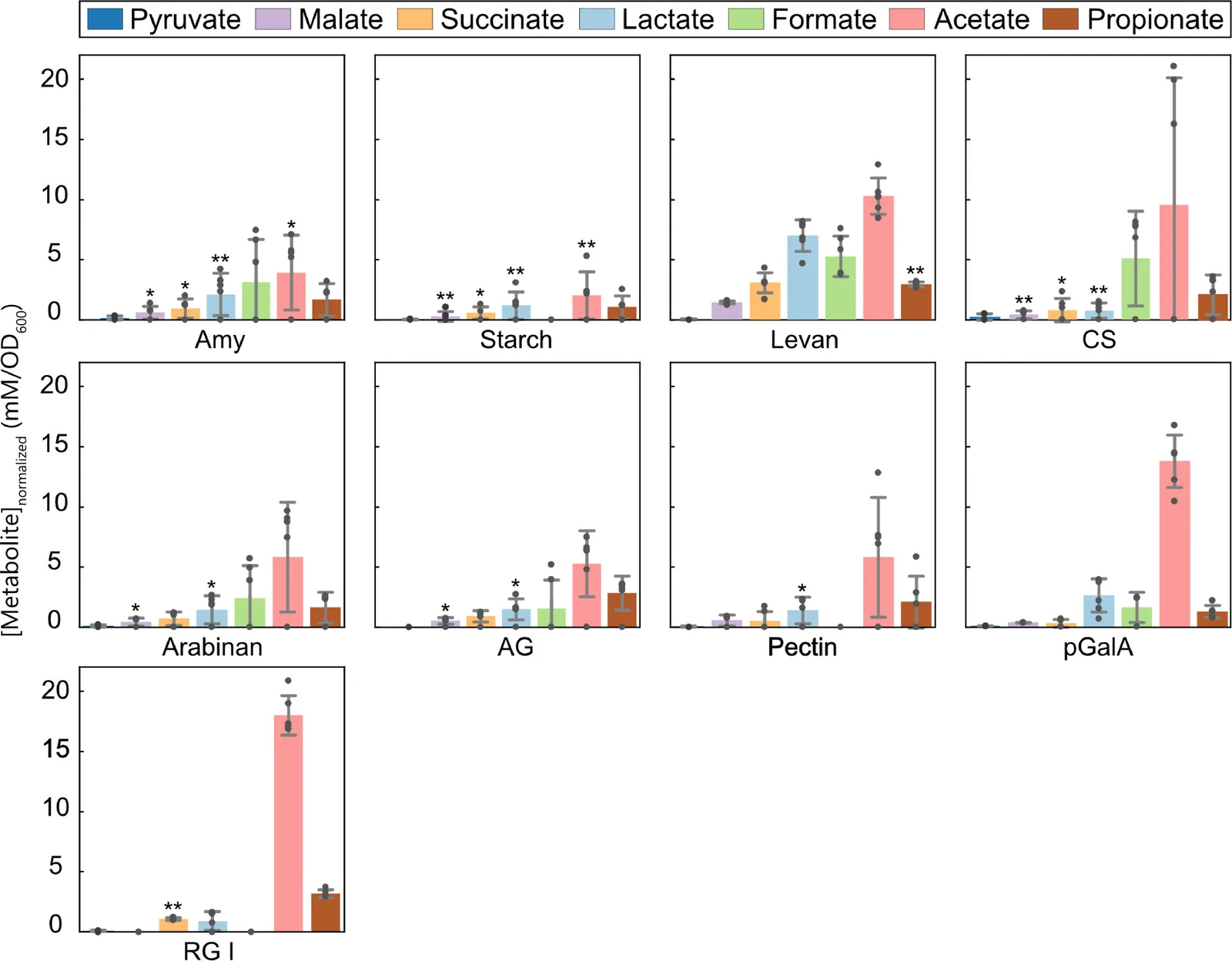
Extracellular metabolites detected from cultures of *Bacteroides thetaiotaomicron* using polysaccharides as carbon sources. The sampling was done as for the monosaccharides; cultures were grown with 5 g/L carbohydrate as the starting carbon source and samples collected at the T_2_ timepoint, and metabolites monitored using HPLC. The concentrations are normalized to the OD_600_ values which were all in a similar range (around 1; Data S1). Individual data points are shown; the bars show the mean value, and error bars represent standard deviation from at least triplicate experiments. Asterisks indicate *P*-values from Student’s T-tests comparing the amount of each metabolite to those produced from their main monosaccharide building block, where possible, with * for *P* ≤ 0.01 and ** for *P* ≤ 0.001. Amylopectin, starch, and CS were compared to Glc; levan to Fru; arabinan to Ara; AG and citrus pectin to Gal; pGalA and RG I to GalA.

The metabolites produced from the polysaccharides were both as expected and not, based on their respective main monosaccharide constituents. From starch and amylopectin, the profiles were not very similar to Glc despite being structurally related homopolysaccharides of glucose, and curiously they were not similar to each other; for instance, no formate was produced from starch, whereas amylopectin produced formate in similar amounts as in Glc cultures (Fig. 5). Overall, as with the oligosaccharides, the metabolite outputs for these polysaccharides were lower than those for Glc. Again, the lower amounts of metabolites could possibly be linked to an added cellular burden of PUL activation and enzyme synthesis linked to polysaccharide deconstruction and import. Similarly, the profile on arabinan differed from that on Ara, with significantly lower production of malate and lactate. Also, on AG, the metabolite amounts were generally lower than those for the Gal culture or any monosaccharide culture. For levan, however, the metabolite profile was similar to that of its Fru building block, and this possibly suggests a more efficient hydrolysis and uptake system for this polysaccharide compared to starch-type ones. The arabinan-targeting PUL, starch utilization system, and fructan-targeting PUL of *B. theta* encode similar numbers of proteins, ten, eight, and nine, respectively (6, 15, 43), but it is possible that the soluble nature of levan reduces the need for protein synthesis to reach a comparable degradation rate compared to that on semi-insoluble starch. Also, pGalA- and RG I-grown cultures resulted in similar metabolite profiles compared to their only, or main building block, GalA, indicating again a similar low burden to grow on these polysaccharides as for the monosaccharide despite the much larger pectin-targeting PULs encoded in *B. theta,* which include well over 50 different genes, the majority of which are enzymes (3, 9). Metabolite production was relatively high on CS, with similar acetate production and higher formate production compared to Glc (Fig. 5).

**Fig 6:**
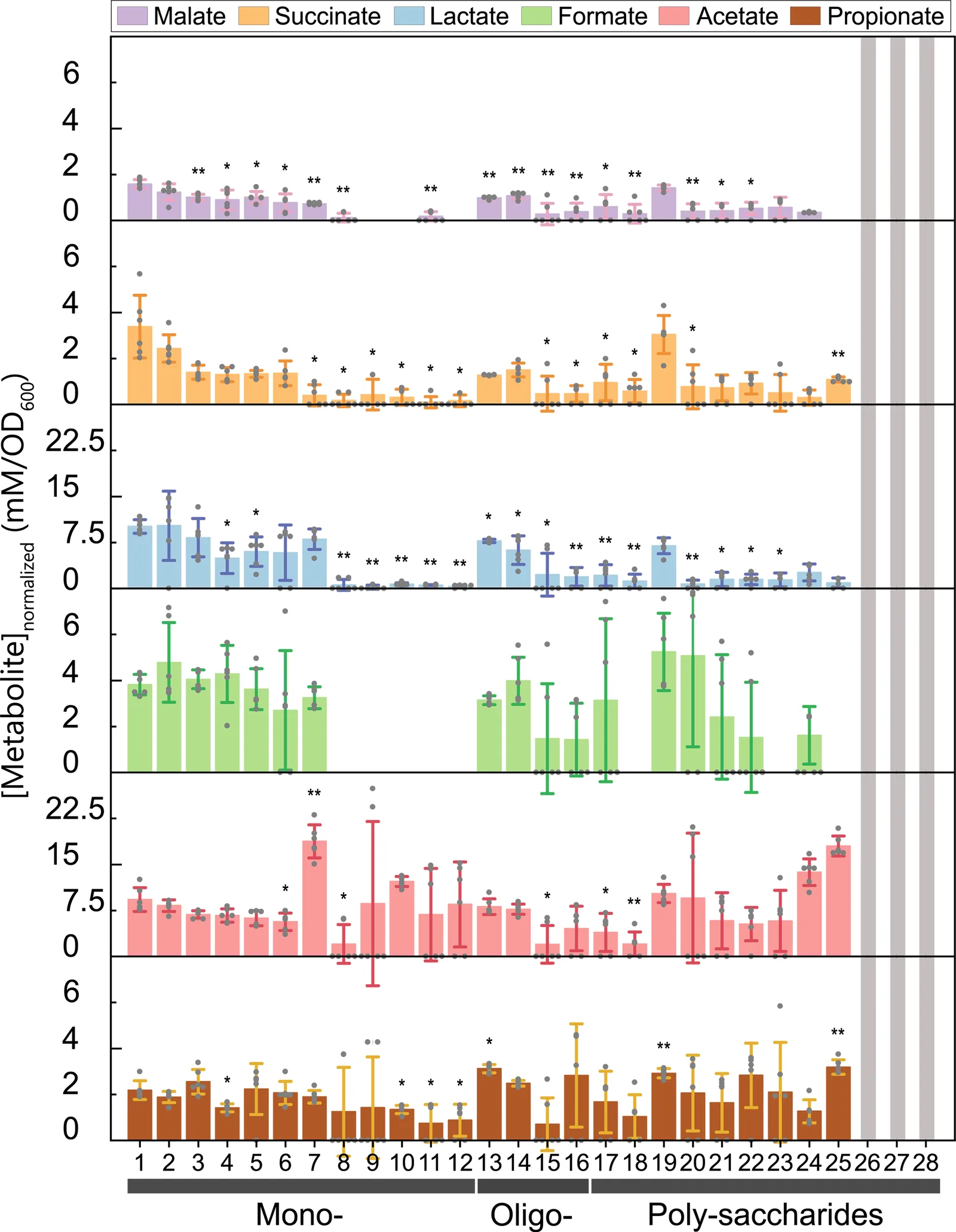
Overall production of OAs from *Bacteroides thetaiotaomicron* cultures with different carbohydrates as carbon source. The samples are sorted by carbohydrate types, and as in Fig. 2, 3 and 5, the metabolite values are normalized to the OD_600_ values at the sampling point. Carbohydrates that did not support growth (hemicelluloses) are marked with light gray bars. The list of carbohydrates is as follows: 1. Glc, 2. Fru, 3. Gal, 4. Man, 5. Ara, 6. Xyl, 7. GlcNAc, 8. GlcN, 9. Fuc, 10. Rha, 11. GalA, 12. GlcA, 13. Lac, 14. Mal, 15. Suc, 16. Raf, 17. amylopectin, 18. starch, 19. levan, 20. CS, 21. arabinan, 22. AG, 23. citrus pectin, 24. pGalA, 25. RG I, 26. galactomannan, 27. konjac glucomannan, and 28. xyloglucan. Asterisks indicate *P*-values from Student’s T-tests comparing the amount of each metabolite to what was produced from Glc for mono- and oligosaccharides and to the main building block for polysaccharides, with * for *P* ≤ 0.01 and ** for *P* ≤ 0.001, as in Fig. 2 and 3.

Taken together, the collected results demonstrate that the production of extracellular metabolites from carbohydrate metabolism of *B. theta* (at the highest growth OD_600_) is variable (Fig. 6). Acetate and lactate were the main metabolites produced, except on starch, but the measured concentrations could vary for different carbon sources by an order of magnitude. The other monitored and detected metabolites similarly varied greatly depending on the carbon source, but generally reached a lower final concentration, which, in some cases, can be explained by a requirement to synthesize a range of different degradative enzymes and sugar capture-and-transport proteins (2). Even if not accounting for the cell number, i.e., not normalizing to OD_600_, similar trends are seen as the final density of the cultures was overall in a similar range (Table S1).

### Extracellular metabolite production from growth on carbohydrates by *Bacteroides ovatus* and *Bacteroides cellulosilyticus*

**Fig 7:**
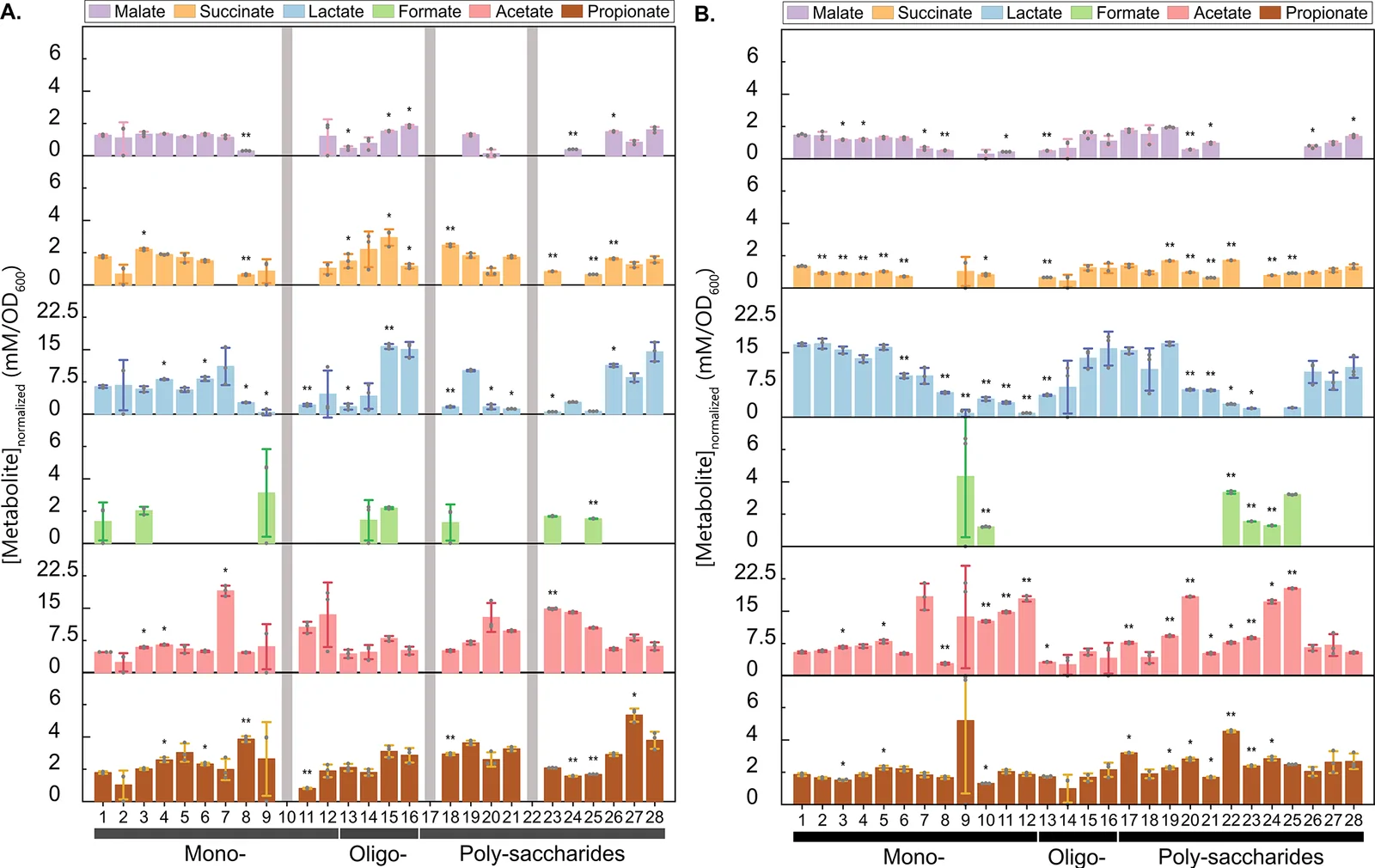
Metabolites detected in carbohydrate-grown cultures of *Bacteroides ovatus* (**A**) and *Bacteroides cellulosilyticus* (**B**). The same metabolite monitoring strategy was used as for *B. theta*, and the carbohydrates are sorted as in Fig. 6, with carbohydrates that did not support growth (hemicelluloses) marked with light gray bars. Carbohydrates used as carbon sources: 1. Glc, 2. Fru, 3. Gal, 4. Man, 5. Ara, 6. Xyl, 7. GlcNAc, 8. GlcN, 9. Fuc, 10. Rha, 11. GalA, 12. GlcA, 13. Lac, 14. Mal, 15. Suc, 16. Raf, 17. amylopectin, 18. starch, 19. levan, 20. CS, 21. arabinan, 22. AG, 23. citrus pectin, 24. pGalA, 25. RG I, 26. galactomannan, 27. konjac glucomannan, and 28. xyloglucan. Asterisks indicate *P*-values from Student’s T-tests comparing the amount of each metabolite to what was produced from Glc for mono- and oligosaccharides and to the main building block for polysaccharides, with * for *P* ≤ 0.01 and ** for *P* ≤ 0.001, as in Fig. 2 and 3. Galactomannan and konjac glucomannan were compared with Man.

To put the results from the *B. theta* cultures into a larger context, similar analyses from mono-, oligo-, and polysaccharide-grown cultures were then conducted for the related species *B. ovatus* and *B. cellulosyliticus* (Fig. 7; Data S1). These species encode different PULs compared to *B. theta* (44), and can additionally grow on hemicellulosic polysaccharides. *B. cellulosyliticus* grew on all carbohydrates tested, while *B. ovatus* was unable to grow on Rha, amylopectin, and AG (Table S1). The final OD_600_ was similar to that of *B. theta* except for a few conditions, with OD_600_ values below 1 for *B. ovatus* on Fuc, starch, arabinan, and RG I, and for *B. cellulosyliticus* on Fuc, amylopectin, AG, and RG I. Overall, the growth of both species resulted in similar ranges of metabolites produced compared to *B. theta*, mainly lactate and acetate, and lower amounts of the other detected OAs, but with different profiles.

*B. ovatus,* in general, produced less diverse OA profiles compared to *B. theta*, where malate, formate, and succinate were not at all detected or detected at trace levels during growth on many carbohydrates, including both mono- and polysaccharides. As for *B. theta*, the profiles for neutral hexoses and pentoses were generally similar to that of Glc, but while *B. theta* also had similar profiles for oligosaccharides; this was not the case for *B. ovatus,* where there was a higher production of lactate than on Glc on both sucrose and raffinose (*P*-value 0.012). On starch, the profile was dissimilar to that on Glc, with a low production of lactate yet higher succinate and propionate levels. As for *B. theta*, the profiles of Fru and levan, and GalA and pGalA/pectin were similar, but interestingly, the profiles of *B. ovatus* from the cultures grown on mannan-based hemicelluloses, gluco- and galactomannan, were not similar to that of Glc/Man/Gal. On glucomannan, a noticeably higher concentration of propionate was observed, and for galactomannan, a higher concentration of lactate than on the constituent monosaccharides. On xyloglucan, which mainly consists of glucose and xylose, there interestingly was a higher production of both lactate and propionate (both with *P*-values of 0.02), contrasting the general observation on *B. theta* with lower metabolite production from polysaccharides. Overall, there was a comparable amount of metabolites produced from growth on these hemicelluloses to that on the monosaccharides, but on the pectic polysaccharides, the amounts were similar to GalA low in general, except acetate and propionate, possibly indicating a more efficient metabolism of the former polysaccharide types for this species.

*B. cellulosilyticus* cultures also displayed a lack of metabolites from certain conditions, similar to *B. ovatus*, with formate in particular being absent in most conditions. The species showed results similar to those of *B. theta* when comparing Glc-grown cultures to those on other neutral hexoses, pentoses, and oligosaccharides, and also on both starch, amylopectin, CS, as well as the hemicelluloses. The clear standout monosaccharide, relative to the metabolite output, was Fuc, with a variable though sporadic much higher production of formate, propionate, and acetate compared to Glc. However, as the growth on this substrate was overall poor, as mentioned previously, these results may instead reflect cell stress. Interestingly, acetate production was higher on GlcA and GalA than on Glc, and also on pGalA and RG I than on GalA; these pectic polysaccharides also led to formate production. Why uronic acid-based carbohydrates lead to different metabolite profiles is unclear, but could potentially be investigated deeper using comparative transcriptomics analyses.

### Conclusion and outlook

Members of the Bacteroidota phylum, and *Bacteroides* strains in particular, have, for several years, been studied in terms of which complex carbohydrates they can deconstruct and metabolize, and especially which PULs they use in the process (2). This has led to the discovery of new enzymes and entire enzyme families (9, 45), but the link to what the organisms produce as a result of carbohydrate catabolism has not received as much attention. Collectively, our results showcase that the output of extracellular metabolites produced by three common gut *Bacteroides* species varies depending on the carbohydrates utilized as carbon sources. This is in contrast to the recent study on the related Bacteroidota member *P. vulgatus*, where the metabolite output was consistent during growth on various carbohydrates (27). The range of carbohydrates used in that study was, however, smaller than the ones employed here, and *P. vulgatus* did not grow on all of them: clear growth was observed on Glc, Fru, Gal, Xyl, Suc, Mal, and Lac, while the strain grew poorly on starch, reaching only approximately half the final OD_600_ as on the former carbohydrates, and not at all on GalA, pGalA, inulin (β[2→1]-linked fructan). The strain was also grown to similar OD_600_ values on Pectin A, but without more detailed information on its composition, it is challenging to link this growth to a particular polysaccharide, as the strain did not grow on either pGalA or GalA, which reportedly would be major components of this substrate. The (non-pectin) carbohydrates that sustained growth for *P. vulgatus* were thus the same as those that resulted in highly similar OA profiles for the *Bacteroides* strains (Fig. 6 and 7). *P. vulgatus* encodes 67 predicted PULs (44), and it is possible that expanding the carbohydrate carbon source range would lead to similar varied OA profiles as in the present work. In another recent study on the Bacteroidete *Segatella copri*, which was grown on Glc and a range of polysaccharides, the metabolite output was similar to *P. vulgatus*; overall quite static across the carbon sources (46). The study, however, reports higher acetate production from pectic polysaccharides, similar to our observations, suggesting similar pathways in the metabolism of these polysaccharides and constituent monosaccharides.

Overall, we did not observe a general correlation between the complexity of the carbohydrate and the metabolite output, i.e., a lower overall output for complex polysaccharides compared to monosaccharides, as the former require a wide range of enzymatic activities for breakdown into individual building blocks that go into central metabolism. A better understanding of the pathways leading to the production of diverse metabolites in these *Bacteroides* species, for instance, via comparative transcriptomics as mentioned previously, and ideally validated in knock-out studies such as those performed with *B. theta* (17), would further shed light on the molecular processes leading to the variable output as shown here. Pure carbohydrates, such as those used here, are not normally encountered in natural environments, and instead diverse compositions will exist and depend both on the source and degradative systems. For instance, plant cell walls that make up dietary fibers will differ substantially depending on species and even tissue (47). How different metabolic pathways of resulting monosaccharides are co-regulated, e.g., for neutral and uronic acids, when catabolized simultaneously from complex glycans would further shed light on their physiology. Similarly, *Bacteroides* species have been shown to have “hard-wired” preference lists for carbohydrates, which determine their nutritional niches (12). How or if this is reflected in metabolic output when grown on mixtures of different carbohydrates should further be investigated. Additionally, how a strain behaves if engineered to grow on new glycans, for example, by transfer of entire PULs (48, 49), remains to be evaluated.

The gastrointestinal tract is densely populated (50), and *in vitro* studies may not reflect the actual behavior of *Bacteroides* species in the gut environment. Many metabolites produced by gut bacteria are well known to have beneficial effects on the host, from providing energy to acting as signaling transmitters (20, 51). Produced metabolites may also be consumed or converted by other species in the gut, e.g., conversion of lactate to butyrate (52), and such microbial interactions may be further affected by different species occupying different layers of the gut, from the lumen to the mucus layer (53). Co-culture experiments with *Bacteroides* and *Bifidobacterium* species have indeed shown effects on metabolite production levels depending both on which strains were combined and also on varied carbohydrates (Glc, inulin, and Bifidobacterial exopolysaccharides) as carbon sources (54). The myriad of interactions and exchange of metabolites between gut species as well as the host cells is a dynamic process, and our study indicates that the metabolite output from a single species is not necessarily static but can vary with something as fundamental as the carbohydrates they use as carbon sources. This has implications for both understanding the composition of the gut microbiota and the effect of diet on human health.

## SUPPLEMENTAL MATERIAL

Data Set S1 aem.00542-26-s0001.xlsx Raw and transformed data.

Supplemental material aem.00542-26-s0002.pdf Fig. S1 and S2; Tables S1 to S3.

ASM does not own the copyrights to Supplemental Material that may be linked to, or accessed through, an article. The authors have granted ASM a non-exclusive, world-wide license to publish the Supplemental Material files. Please contact the corresponding author directly for reuse.
