# Supplementary material for: Extracellular metabolite production by Bacteroides strains varies depending on carbohydrates used as carbon sources
Source: Appl Environ Microbiol. 2026 Jul 21;92(8):e00542-26. doi: 10.1128/aem.00542-26 (PMC13488361; doi:10.1128/aem.00542-26)
Supplement: Supplementary file 1 [file aem.00542-26-s0002.pdf]

Supplemental information for:

Extracellular metabolite production by *Bacteroides* strains varies depending on carbohydrates used as carbon sources

Yi-Hsuan Lee[1], Verena Siewers[2], Johan Larsbrink[1,*]

[1] Division of Industrial Biotechnology, Department of Life Sciences, Chalmers University of Technology, SE-412 96, Gothenburg, Sweden

[3] Division of Systems and Synthetic Biology, Department of Life Sciences, Chalmers University of Technology, SE-412 96, Gothenburg, Sweden

[*] correspondence: johan.larsbrink@chalmers.se

Supplementary Figures

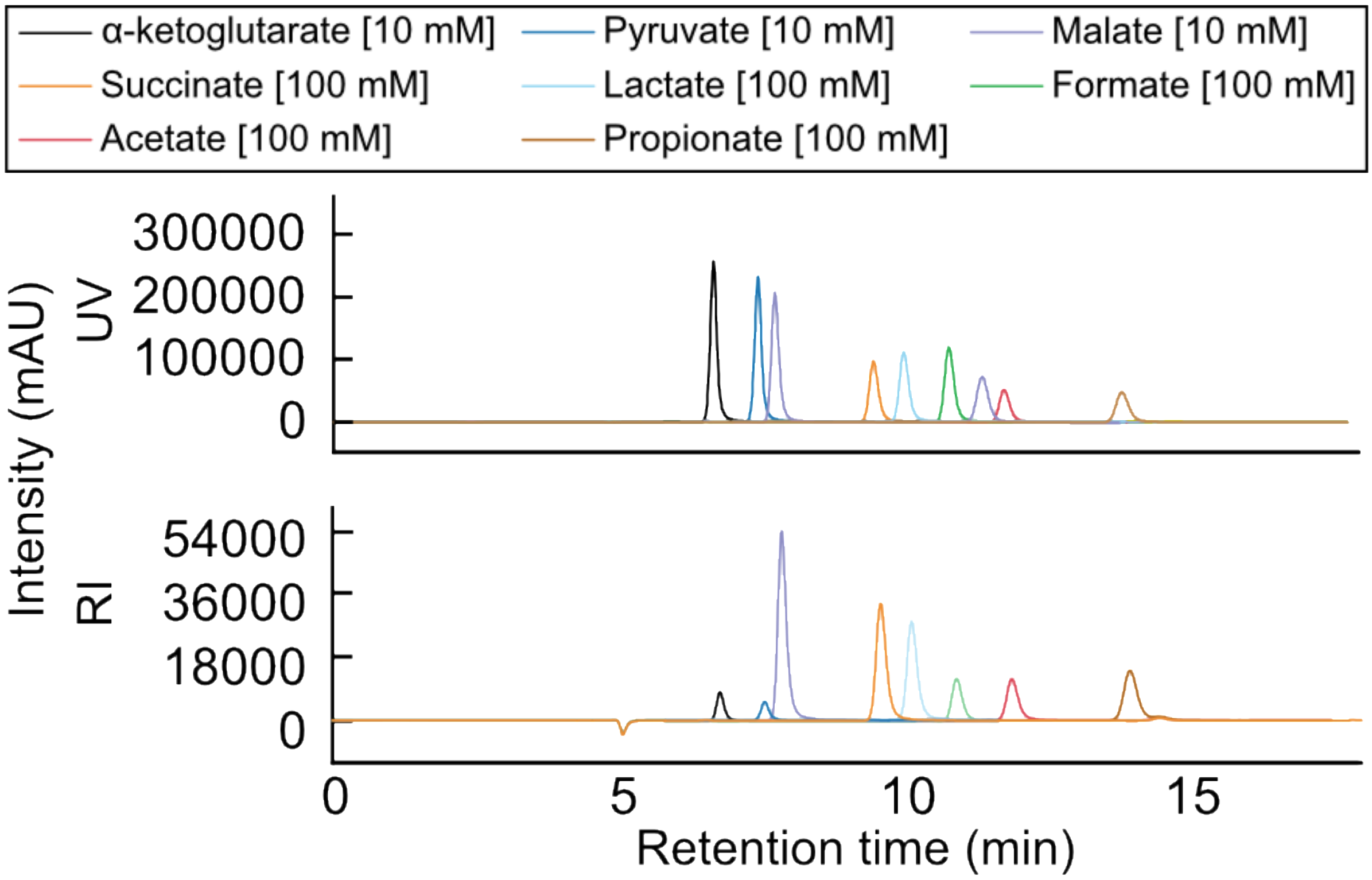

Figure S1. HPLC chromatograms of metabolites sampled in *Bacteroides* cultures. The metabolite standards are shown, both with UV (top) and refractive index (RI; bottom) detection. Note that in the malate standard, a small amount of a compound of unknown identity was present (~12 min).

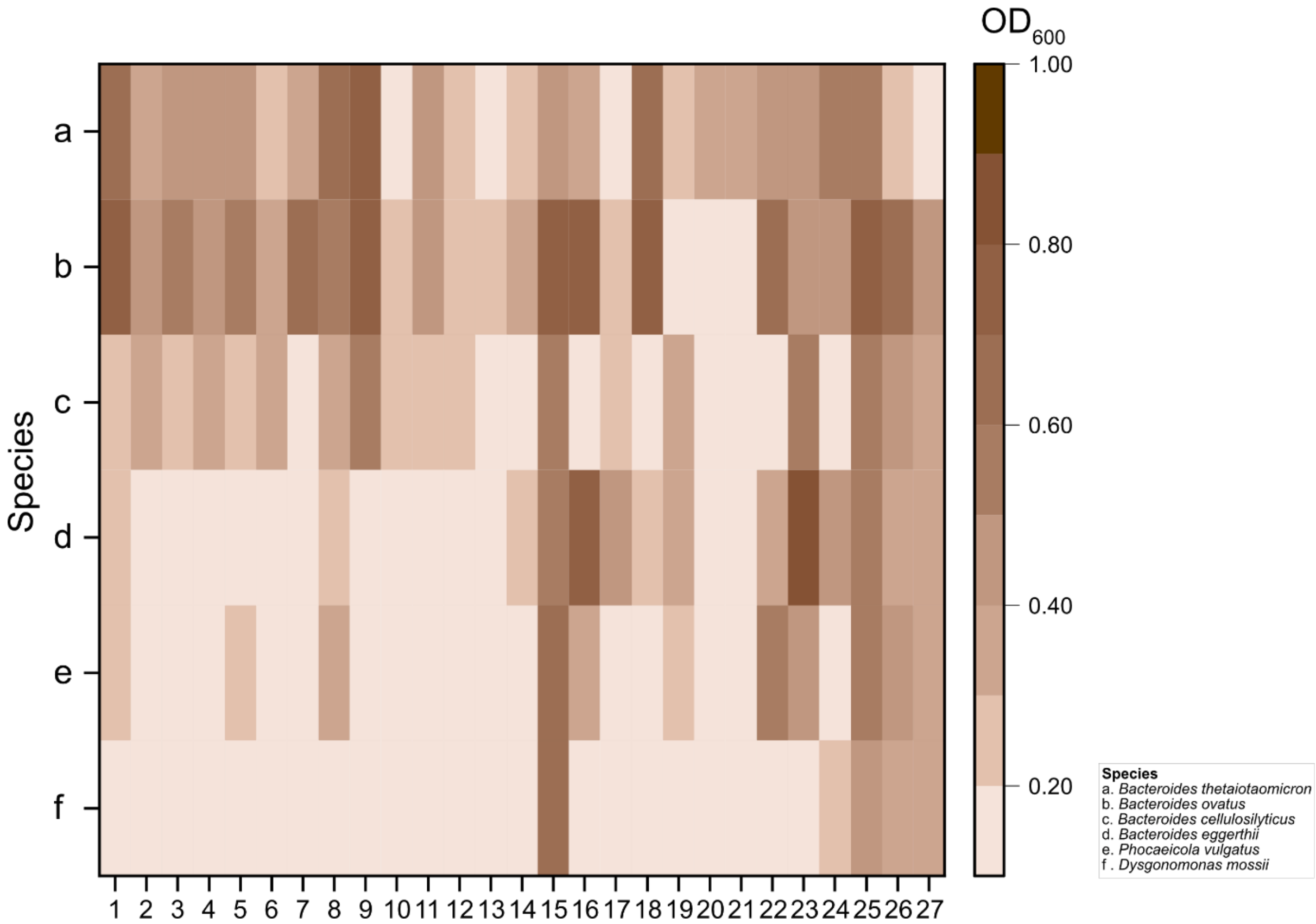

Figure S2. Growth coupled to carbohydrate utilization by six chosen Bacteroidota species. Six selected Bacteroidota species were tested: *B. thetaiotaomicron*, *B. ovatus*, *B. cellulosilyticus*, *B. eggerthii*, *P. vulgatus*, and *D. mossii*. The heatmap shows the overall growth on a range of selected carbohydrates used as the sole carbon source, from monosaccharides to more polysaccharides. The OD_{600} data are based on triplicate analyses. The numeric list of carbohydrates is as follows: 1. Glc, 2. Ara, 3. Fru, 4. Gal, 5. GalA, 6. GlcNAc, 7. GlcN, 8. GlcA, 9. Man, 10. Rha, 11. Xyl, 12. lactose, 13. maltose, 14. sucrose, 15.raffinose, 16. amylopectin, 17. starch, 18. levan, 19. chondroitin sulfate, 20. arabinan, 21. arabinogalactan, 22. citrus pectin, 23. pGalA, 24. RG I, 25. galactomannan, 26. konjac glucomannan, and 27. xyloglucan.

Supplementary Tables

Table S1. Maximum OD_{600} reached for the three investigated *Bacteroides* species, and the time to reach this maximum. The data are based on at least triplicate samples from 2-mL tube cultures and errors represent standard deviation. Dashes signify no measurable growth.

	Bacteroides thetaiotaomicron		***Bacteroides ovatus***		***Bacteroides cellulosyliticus***	
**Carbohydrate**	**Max OD_{600}**	**Time (h)**	**Max OD_{600}**	**Time (h)**	**Max OD_{600}**	**Time (h)**
Glucose	1.58 ± 0.017	33.0	1.68 ± 0.017	24.5	1.47 ± 0.042	29.5
Arabinose	1.75 ± 0.034	21.5	1.49 ± 0.047	41.0	1.29 ± 0.022	29.5
Fructose	1.42 ± 0.026	31.0	1.47 ± 0.062	20.5	1.43 ± 0.008	21.0
Fucose	1.06 ± 0.036	39.5	0.52 ± 0.015	34.5	0.54 ± 0.021	27.0
Galactose	1.55 ± 0.020	23.5	1.91 ± 0.016	15.0	1.51 ± 0.024	21.0
GalA	1.32 ± 0.022	14.5	1.48 ± 0.041	24.5	1.36 ± 0.010	40.5
GlcNAc	1.66 ± 0.043	20.0	1.72 ± 0.025	22.0	1.42 ± 0.020	23.5
GlcN	1.23 ± 0.017	36.5	1.78 ± 0.005	44.0	1.65 ± 0.005	41.5
GlcA	1.50 ± 0.017	33.5	1.52 ± 0.054	31.0	1.08 ± 0.015	49.5
Mannose	1.80 ± 0.005	20.0	1.62 ± 0.039	20.5	1.46 ± 0.010	26.5
Rhamnose	1.11 ± 0.035	32.5	-	-	1.06 ± 0.016	21.0
Xylose	1.24 ± 0.025	14.0	1.74 ± 0.034	41.0	1.38 ± 0.015	21.0
Lactose	1.65 ± 0.012	38.0	1.83 ± 0.022	20.0	1.81 ± 0.021	21.5
Maltose	1.55 ± 0.006	33.5	1.83 ± 0.005	23.0	1.43 ± 0.022	40.5
Sucrose	1.60 ± 0.042	41.5	1.76 ± 0.010	33.5	1.47 ± 0.029	21.5
Raffinose	1.63 ± 0.021	94.0	1.36 ± 0.012	123.0	1.41 ± 0.038	32.5
Amylopectin	1.42 ± 0.012	46.0	-	-	0.87 ± 0.033	21.5
Starch	1.37 ± 0.021	46.0	0.73 ± 0.170	33.5	1.15 ± 0.035	40.5
Levan	1.57 ± 0.025	33.0	1.63 ± 0.041	24.0	1.59 ± 0.008	27.5
Chondroitin sulfate	1.14 ± 0.041	24.0	1.27 ± 0.005	30.0	1.13 ± 0.056	29.5
Arabinan	1.05 ± 0.024	20.0	0.69 ± 0.054	33.5	1.46 ± 0.010	45.0
Arabinogalactan	1.04 ± 0.038	25.0	-	-	0.43 ± 0.031	27.5
Citrus pectin	1.52 ± 0.027	41.5	1.38 ± 0.008	23.0	1.13 ± 0.025	21.0
RG I	1.54 ± 0.008	37.0	0.86 ± 0.012	24.0	0.82 ± 0.033	21.0
pGalA	1.52 ± 0.017	14.5	1.15 ± 0.019	22.0	1.21 ± 0.008	15.5
Galactomannan	-	-	1.53 ± 0.012	32.0	1.41 ± 0.034	50.5
Konjac glucomannan	-	-	1.56 ± 0.008	32.0	1.13 ± 0.005	93.5
Xyloglucan	-	-	1.14 ± 0.007	31.0	1.13 ± 0.020	114.5

Table S2. Remaining carbohydrates at the end of *Bacteroides* cultivation, in g/L, as determined using HPAEC-PAD. For cultures on oligosaccharides, both the initial carbon source (i.e., oligosaccharides, 5 g/L) and the constituent monosaccharides were quantified, as indicated by dashed lines. The data are average quantities measured from triplicate samples, where a carbohydrate is considered detected when identified in at least two replicates, with errors representing standard deviation.

Carbohydrate	*Bacteroides thetaiotaomicron*	*Bacteroides ovatus*	*Bacteroides cellulosilyticus*
Arabinose	0.0227±0.02	0.0272±0.01	0.0287±0.02
Fructose	0.3008±0.21	ND	0.0436±0.00
Galactose	0.0877±0.06	0.0040±0.00	0.0244±0.00
Glucose	0.0102±0.00	0.0014±0.00	0.0116±0.00
Mannose	0.0440±0.02	0.0012±0.00	0.0186±0.00
Rhamnose	0.0352±0.02	-	0.1409±0.00
Xylose	0.0503±0.00	0.1339±0.04	0.0083±0.01
Lactose	0.1026±0.08	0.1593±0.10	0.0321±0.01
– Galactose	0.0010±0.00	ND	ND
– Glucose	0.0009±0.00	0.0006±0.00	ND
Maltose	0.0070±0.00	0.0106±0.01	0.0118±0.01
– Glucose	ND	ND	ND
Raffinose	0.1813±0.10	0.0159±0.00	0.0015±0.00
– Fructose	0.0617±0.00	ND	0.0017±0.00
– Galactose/Glucose*	0.0056±0.00	ND	0.0051±0.00

ND, not detected. - No growth.
*Raffinose and its products were analyzed using a PA200 column where Gal and Glc co-elute.

Table S3. pH measured at timepoint T_0 and T_3 alongside the *B. thetaiotaomicron* growth.

pH in cultures with carbohydrate	T_0	T_3
Bacteroides Minimal Medium	6.91	5.90
Glucose	6.90	5.76
Arabinose	6.92	5.83
Fructose	6.92	6.15
Fucose	6.94	6.45
Galactose	7.12	5.54
GalA	6.89	6.75
GlcNAc	7.02	6.62
GlcN	6.72	5.51
GlcA	6.99	6.66
Mannose	6.91	5.41
Rhamnose	6.95	6.55
Xylose	6.94	6.41
Lactose	7.06	6.68
Maltose	6.41	5.59
Sucrose	6.92	5.58
Raffinose	6.99	6.15
Amylopectin	6.90	5.60
Starch	6.75	6.51
Levan	6.93	5.43
Chondroitin sulfate	6.90	7.04
Arabinan	6.63	6.62
Arabinogalactan	6.90	6.74
Citrus pectin	6.82	6.53
RG I	6.97	6.20
PGalA	6.86	6.73
Galactomannan	6.80	6.20
Konjac glucomannan	6.89	5.91
Xyloglucan	6.90	6.11
